# Local–Global Aware Concept Bottleneck Models for Interpretable Image Classification

**DOI:** 10.3390/s26061833

**Published:** 2026-03-14

**Authors:** Ci Liu, Zijie Lin, Chen Tang

**Affiliations:** School of Electrical and Information Engineering, Tianjin University, Tianjin 300072, China; liuci@tju.edu.cn (C.L.); linzijie@tju.edu.cn (Z.L.)

**Keywords:** interpretable artificial intelligence, concept bottleneck models, image classification, few-shot learning

## Abstract

Concept Bottleneck Models facilitate interpretable image classification by predicting human-understandable concepts prior to class labels. However, when constructed upon CLIP, they exhibit unreliable concept scores stemming from CLIP’s global representation bias and insufficient region-level sensitivity, which severely constrain their effectiveness in sensor-driven applications like remote sensing and medical imaging where localized visual evidence is critical. To mitigate this, we propose the Local–Global Aware Concept Bottleneck Model (LGA-CBM), which improves concept prediction through a training-free refinement pipeline. Building on initial CLIP-derived concept scores, LGA-CBM incorporates three key components: a Dual Masking Guided Concept Score Refinement (DMCSR) module that exploits attention weights to strengthen region–concept alignment; a Local-to-Global Concept Reidentification (L2GCR) strategy to harmonize local and global activations; and a Similar Concepts Correction Mechanism (SCCM) integrating Grounding DINO for fine-grained disambiguation. A sparse linear layer then maps the refined concepts to class labels, enabling highly interpretable classification with minimal concept usage. Experiments across six benchmark datasets demonstrate that LGA-CBM consistently achieves state-of-the-art performance in both accuracy and interpretability, producing explanations that align closely with human cognition.

## 1. Introduction

Accurate and reliable visual understanding is a fundamental requirement in sensor-driven intelligent systems, including remote sensing interpretation [1,2], medical image analysis [3,4], and intelligent monitoring [5,6]. In such applications, visual perception modules are responsible for extracting high-level semantic information from complex sensor data, directly influencing system robustness and decision reliability.

The rapid evolution of image-based sensing systems has been largely driven by advances in deep learning models, which have continuously improved the accuracy and generalization capability of visual recognition. Early breakthroughs in image classification were primarily achieved through convolutional neural networks (CNNs), which established strong performance baselines by enhancing feature extraction and representation learning. Representative architectures such as ResNet [7] and DenseNet [8] improved gradient propagation and feature reuse, while HCGNet [9] introduced gated mechanisms to strengthen adaptive feature modeling. Building upon these structural innovations, subsequent research further advanced visual representation learning across diverse sensing environments. More recently, transformer-based architectures and vision–language pretraining paradigms have expanded the modeling capacity of visual systems, enabling cross-modal semantic alignment and large-scale representation learning.

Despite the substantial improvements in recognition performance brought by deep learning models, most deep architectures remain inherently black-box, with decisions derived from high-dimensional embeddings that lack explicit semantic interpretability. Such opacity poses significant challenges in safety-critical and high-stakes sensing applications, where transparent reasoning and trustworthy perception are indispensable [10,11,12,13,14]. Consequently, enhancing interpretability alongside predictive performance has become a crucial research direction in sensor-based visual intelligence.

Concept-based explainability methods have emerged as a promising direction to bridge the gap between model decisions and human cognition. Inspired by the manner in which humans comprehend the world through abstract and semantically meaningful concepts rather than low-level sensory signals, these methods aim to elucidate model behavior by mapping inputs and predictions to a finite set of interpretable concepts. Early studies investigated the relationship between internal neural representations and visual semantics by associating neuron activations with predefined visual concepts, revealing how convolutional neural networks encode semantic information at varying levels of abstraction [15,16]. Subsequent works further advanced this paradigm by proposing automatic concept discovery and attribution methods, including the unsupervised clustering of activation maps [17,18], attention-guided part selection mechanisms [19], and gradient-based concept importance measures [20]. These approaches significantly improved the transparency of deep models by elevating explanations from pixel-level responses to the semantic concept level.

Compared to other concept-based explanation methods, Concept Bottleneck Models (CBMs) [21] provide a more structured and intervention-friendly framework for interpretability. By explicitly introducing a concept bottleneck layer into the model architecture, CBMs mandate that all predictions pass through a set of human-understandable concepts, enabling not only explanation but also direct manipulation and debugging of model behavior. This architectural constraint grants CBMs unique advantages in model transparency, model editing, and human–AI interaction [21,22,23]. Early CBMs relied heavily on expert-defined concept annotations, which, while precise, were hindered by high annotation costs and limited scalability, particularly in large-scale or domain-specific datasets.

Recent advances in large language models and vision–language models have significantly transformed the construction of CBMs. By leveraging large language models to automatically generate semantically rich concept sets and employing CLIP-style models to compute image–concept similarities, modern CBMs have largely obviated the dependency on manual concept annotation [24,25,26]. Representative works such as LM4CV [26] and LaBo [25] utilize CLIP [27] to project images and textual concepts into a shared embedding space, where cosine similarity serves as the concept score. These developments have greatly improved the scalability of CBMs and enabled their application to large-scale datasets.

However, despite these advances, a critical yet underexplored issue remains in CLIP-based CBMs: inaccurate and unreliable concept prediction. Even with high-quality concept sets generated by large language models, correctly mapping images to their corresponding concepts remains challenging. Most existing CBMs directly employ cosine similarity between image and text embeddings as the concept activation score, implicitly assuming that CLIP embeddings provide sufficient semantic discrimination at the concept level. In practice, this assumption often proves invalid, particularly in fine-grained recognition tasks or sensor-based scenarios where multiple visual concepts co-occur and jointly support decision-making.

Human cognition typically associates an object category with multiple semantically relevant concepts. Accordingly, a well-functioning CBM is expected to yield strong responses for concepts present in an image while suppressing those that are absent. However, many existing CBMs fail to exhibit this behavior in practice. When cosine similarity between image and text embeddings is directly employed as the concept score, the resulting activations across different textual concepts tend to be uniformly low and poorly discriminative. This phenomenon stems from the reliance on CLIP’s frozen image and text encoders, which project visual inputs and textual concepts into a shared embedding space. During CLIP pretraining, the class token in the image encoder is primarily optimized to capture global image representations, yielding limited sensitivity to region-level or part-level concept cues. From a sensing perspective, such a global representation paradigm hampers CLIP’s effectiveness as a semantic perception module since many visual concepts are inherently linked to localized sensory evidence rather than holistic appearance features.

An alternative strategy leverages CLIP’s zero-shot classification framework to reshape the concept score distribution. However, the application of the Softmax function typically amplifies a single dominant concept while suppressing other relevant ones. As a result, only one textual concept exhibits a strong response, even when multiple concepts are clearly present in the image. This limitation not only yields concept explanations that deviate from human cognitive understanding but also diminishes the reliability of CBMs in practical perception scenarios, such as fine-grained visual recognition in remote sensing, medical image analysis, and intelligent monitoring systems, where multiple co-occurring concepts jointly support robust decision-making.

**Figure 1:**
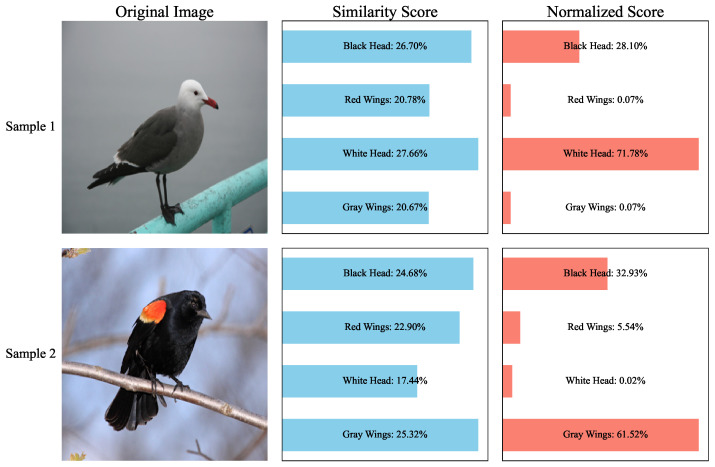
Issues with conventional concept score calculation methods.

As illustrated in Figure 1, two bird images from different classes in the CUB-200 [28] dataset are selected, and four visual concepts are considered, namely black head, red wings, white head, and gray wings. Sample 1 is primarily associated with white head and gray wings, whereas Sample 2 corresponds to black head and red wings. Concept scores are computed using CLIP ViT-B/32. When cosine similarity is directly employed as the concept score, as in LM4CV, the similarity values of concepts that are clearly relevant to a given sample do not exceed 0.3 and exhibit little distinction from those of irrelevant concepts, indicating limited discriminative capability in concept activation.

In contrast, when the concept scoring strategy proposed in LaBo is utilized, where cosine similarities are scaled by a factor of 100 and normalized using the Softmax function, only a single concept yields a strong response. This behavior conflicts with the established understanding that an object is typically associated with multiple visual concepts. Furthermore, due to the limited ability of CLIP to capture region-level semantic information, errors introduced during the similarity computation stage are further amplified by the Softmax normalization, leading to more severe concept misidentification. The limitation of CLIP in recognizing image regions has been documented in prior studies. RegionCLIP [29] points out that CLIP pretraining focuses on global image–text matching without enforcing fine-grained alignment between image regions and textual descriptions. Subsequent works have sought to mitigate this issue through region-aware pretraining, knowledge distillation, or region–text alignment strategies [29,30,31,32,33,34]. However, within the context of Concept Bottleneck Models, the impact of CLIP’s limited region-level semantic perception on concept score reliability has received insufficient attention. This deficiency directly undermines the validity of concept prediction, complicates downstream concept reasoning, and ultimately degrades both classification performance and interpretability.

From the perspective of image-based sensing systems, reliable concept perception is not only a matter of interpretability but also a fundamental requirement for robust sensor intelligence. In many sensor-driven applications, such as remote sensing interpretation, medical imaging, and intelligent monitoring, visual inputs are acquired under complex conditions involving noise, viewpoint variations, and multi-scale structures. In these scenarios, semantic understanding often relies on the aggregation of multiple localized visual cues rather than a single dominant global feature. Consequently, explainable AI models deployed in sensor systems must be capable of accurately perceiving and reasoning over both local and global semantic evidence. However, existing CLIP-based Concept Bottleneck Models, which primarily rely on global image representations, struggle to provide reliable concept activations under such sensing conditions. This limitation directly affects the robustness, transparency, and trustworthiness of AI-powered image sensor systems, highlighting an urgent need for concept-aware perception mechanisms tailored to sensor-based visual understanding.

To address these challenges, we propose a Local–Global Aware Concept Bottleneck Model (LGA-CBM), which improves the reliability of concept activation by jointly modeling local region-level evidence and global visual context. By enhancing the semantic perception capability of CLIP-based backbones without introducing heavy training overhead, LGA-CBM enables more accurate and interpretable concept reasoning for sensor-driven visual understanding tasks.

The main contributions of this work are summarized as follows:

(1) We investigate the limitations of CLIP when utilized as the backbone network in CBMs and demonstrate that the visual features from the final layer are inadequate for reliable concept score computation.
(2) We propose a Dual Masking Guided Concept Score Refinement (DMCSR) module based on the attention weights of CLIP, which strengthens the alignment between image regions and textual concepts without introducing additional training overhead. Furthermore, a Local-to-Global Concept Reidentification (L2GCR) strategy is presented to optimize the activation strengths of both local and global concepts within images.
(3) We develop a Similar Concepts Correction Mechanism (SCCM) by integrating Grounding DINO with CLIP, which mitigates the lack of discriminative ability among similar concepts in fine-grained datasets.
(4) We map the refined concept scores to class labels using a sparse linear layer, achieving state-of-the-art classification performance under interpretable settings across six benchmark datasets, validating the effectiveness of the proposed model for reliable and interpretable visual perception in sensor-based intelligent systems.

## 2. Materials and Methods

### 2.1. Datasets

We utilize multiple publicly available benchmark datasets to validate the effectiveness of LGA-CBM, including CIFAR-10 [35], CIFAR-100 [35], CUB-200 [28], Food-101 [36], Flower-102 [37], and ImageNet-100 [38]. These datasets span a wide range of scenarios from general image classification to fine-grained categorization, providing a solid foundation for comprehensive model evaluation. The following offers a detailed overview of each dataset:

(1) CIFAR-10. CIFAR-10 was introduced in [35] and consists of 60,000 color images of size 32×32 pixels. The dataset contains 10 classes: airplane, automobile, bird, cat, deer, dog, frog, horse, ship, and truck. Each class includes 5000 training images and 1000 test images, ensuring consistent evaluation of model performance during both training and testing phases.
(2) CIFAR-100. Also proposed in [35], CIFAR-100 follows a similar structure to CIFAR-10 but increases the number of classes to 100. Each class contains 500 training images and 100 test images, totaling 60,000 color images of size 32×32 pixels. The 100 classes are grouped into 20 superclasses based on semantic similarity, making this dataset more suitable for evaluating model performance on fine-grained classification tasks.
(3) CUB-200. CUB-200 is a fine-grained bird classification dataset introduced in [28]. It contains 11,788 images across 200 bird species, with 5994 images for training and 5794 for testing. Each image is annotated with 15 keypoint locations and accompanied by detailed textual descriptions of the species, rendering it a key benchmark for fine-grained classification and multimodal learning tasks.
(4) Food-101. Released in [36], Food-101 comprises 101 food categories with a total of 101,000 images. Each category includes 750 training images and 250 test images. The images exhibit high resolution, significant intra-class variation, and complex background clutter, making this dataset well suited for assessing a model’s ability to recognize concepts in challenging real-world scenarios.
(5) Flower-102. Proposed in [37], Flower-102 contains 8189 images of 102 flower species. The dataset is split into 1020 training images, 1020 validation images, and 6149 test images. The visual appearance of flowers exhibits subtle fine-grained differences and rich visual diversity, offering an excellent testbed for fine-grained feature learning.
(6) ImageNet-100. ImageNet-100 is a subset of the ImageNet dataset [38], containing 100 classes with approximately 1300 training images and 50 validation images per class. The selected classes cover a broad distribution ranging from animals to objects and are non-overlapping with other ImageNet subsets. As a lightweight alternative to the full ImageNet, ImageNet-100 enables the efficient evaluation of model performance on high-resolution images.

These datasets collectively provide a diverse set of task environments for evaluating LGA-CBM across multiple scenarios, covering both general and fine-grained classification settings. This diversity ensures a thorough validation of the model’s effectiveness and robustness.

### 2.2. Methods

**Figure 2:**
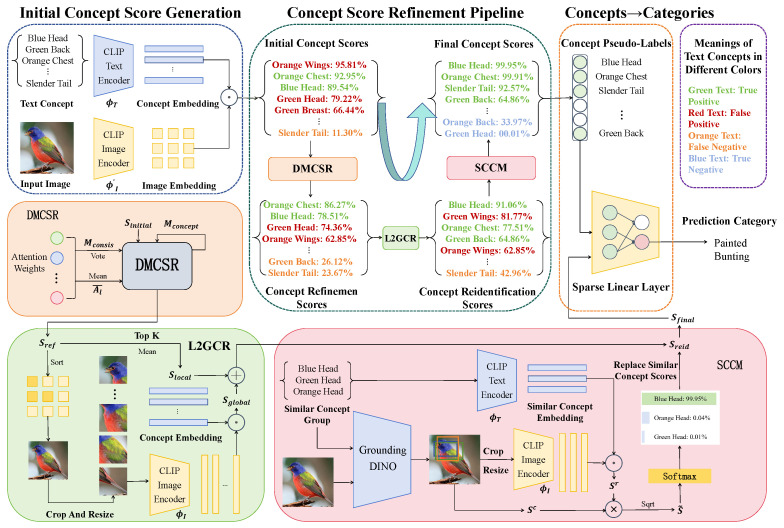
Illustration of the proposed LGA-CBM framework.

This section provides a detailed description of the proposed LGA-CBM. As illustrated in Figure 2, the method consists of three main components: initial concept score generation, concept score refinement pipeline, and interpretable mapping from concepts to class labels. First, we analyze CLIP and conclude that the visual embeddings produced by the original CLIP are unsuitable for direct similarity computation with textual concept embeddings. Therefore, we compute the initial concept scores $S_{\mathrm{initial}}$ using image patch embeddings that contain dense tokens. Second, the concept score refinement pipeline applies three sequential optimization steps through the DMCSR module, the L2GCR module, and the SCCM module. Specifically, (1) the initial concept scores $S_{\mathrm{initial}}$ are processed by the DMCSR module to obtain refined concept scores $S_{\mathrm{ref}}$, which reduces the scores of concepts that do not actually appear in the image (false positives) in dense prediction; (2) the refined scores $S_{\mathrm{ref}}$ are further enhanced by the L2GCR module to strengthen recognition of holistic concepts by integrating local and global concept information, thereby improving the joint detection of both local and global concepts (true positives) and yielding reidentified concept scores $S_{\mathrm{reid}}$; (3) the reidentified scores $S_{\mathrm{reid}}$ are passed through the SCCM module, which selects the most matching concept within each group of similar concepts as the true concept and uses the Softmax function to widen the gap between similar concepts (true positives versus true negatives), resulting in the final concept scores $S_{\mathrm{final}}$ that reflect the true activation strength of each textual concept in the image. Finally, a sparse linear layer maps the final concept scores $S_{\mathrm{final}}$ to class labels, enabling interpretable image classification.

#### 2.2.1. Task Definition

Given an image-label paired dataset D={(x,y)}, where x∈X denotes an image and y∈Y denotes its corresponding label, we define a set of *N* discrete concepts $E=\{e_{1},e_{2},\ldots ,e_{N}\}$ to describe the key semantic information present in the images. These concepts are selected by a predefined procedure and represented in discrete form. In multimodal pretrained models such as CLIP, an image encoder $\Phi_{I}$ and a text encoder $\Phi_{T}$ map images and textual inputs, respectively, into a shared *d*-dimensional feature space.

The text encoder $\Phi_{T}$ encodes each concept $e_{i}$, and the resulting embedding is $L_{2}$ normalized to produce the concept embedding $w_{i}=\Phi_{T}\left(e_{i}\right)$. All concept embeddings collectively form a concept pool, which can be represented as a concept projection matrix $W\in R^{N\times d}$ that maps *d*-dimensional visual features into an *N*-dimensional concept space. For each image *x*, the image encoder $\Phi_{I}$ extracts its visual representation $f=\Phi_{I}\left(x\right)$. Since the image and text feature spaces in CLIP are aligned during pretraining, the visual representation *f* and the concept embeddings $w_{i}$ reside in the same feature space. Concept scores for the image are obtained by computing the dot product c=W·f, where each $c_{i}$ denotes the score of concept $e_{i}$ and indicates its presence in the image.

Most recent CBMs are built upon the pretrained CLIP model. In contrast to the original CBM, they obtain the concept bottleneck by directly projecting the visual representation *f* onto the concept pool *W* rather than learning an additional concept extractor. After selecting relevant concepts, only a linear classifier $\Psi_{c}:R^{N}\to Y$ needs to be learned for image classification. The entire process can be expressed as

(1) $$\hat{y}=\Psi_{c}\left(c\right)=\Psi_{c}\left(W\cdot f\right)$$

#### 2.2.2. Initial Concept Scores Based on Dense Tokens

Current mainstream CBMs typically generate an initial concept set using large language models, subsequently selecting a subset exhibiting higher relevance to target classes via various filtering strategies. These models commonly compute concept logits by measuring image–text concept similarity using CLIP. However, the resulting logit distribution tends to be overly uniform, failing to accurately reflect the relative presence of visual attributes within the image. This frequently leads to the counterintuitive scenario where textual concepts clearly absent from the image receive higher scores than those actually present. Such inaccuracies severely compromise concept score reliability, thereby degrading both classification accuracy and interpretability.

The root cause of this issue stems primarily from the CLIP’s original training paradigm. First, CLIP is trained on image–text pairs using contrastive loss, which aims to match an image with its corresponding textual description while distinguishing it from unrelated texts. The Softmax operation inherent in this loss induces competition among different categories, which is incompatible with the CBM requirement to assign multiple relevant concepts to a single image. Second, CLIP represents the entire image using a single global embedding derived from the class token, without explicitly modeling local features from specific image regions. This design further restricts its ability to capture region-specific concepts within the image.

Given an *L* layer image encoder CLIP-ViT, the forward propagation at the final layer can be expressed as

(2) $$\begin{array}{cc} X^{L} & ={\hat{X}}^{L}+\mathrm{MLP}({\hat{X}}^{L})\\ \; & =X^{L-1}+a^{L}+\mathrm{MLP}(X^{L-1}+a^{L})\\ \; & =X^{L-1}+A^{L}(X^{L-1}W_{V}^{L})+\mathrm{MLP}(X^{L-1}+A^{L}(X^{L-1}W_{V}^{L})), \end{array}$$

where $X^{L-1}$ denotes the output of layer L−1, and $a^{L}$ and MLP represent the self attention and multi-layer perceptron modules in ViT, respectively. The matrix $A^{L}=\sigma \left(\frac{\left(X^{L-1}W_{Q}^{L}\right){\left(X^{L-1}W_{K}^{L}\right)}^{T}}{\sqrt{d}}+M^{L}\right)$ encodes the attention weights at layer *L*, where σ denotes Softmax normalization, *d* is the dimensionality of $X^{L-1}$, $M^{L}$ is the attention mask, and $W_{Q}$, $W_{K}$, and $W_{V}$ are the linear projection matrices used in multihead self attention to produce query, key, and value vectors, respectively. The output $X^{L}$ consists of a class token and dense tokens, which can be written as

(3) $$X^{L}=\left[x_{\mathrm{cls}}^{L},x_{\mathrm{dense}}^{L}\right].$$

Many studies argue that removing the class token from the final layer output of the CLIP image encoder enhances its ability to capture region-level features. Ghiasi et al. [39] observed that the class token plays a negligible role throughout most layers of ViT and is employed only in the final layer to extract global image information, thus leaving the local features extracted in the first L−1 layers unaffected. Lin et al. [40] further noted that in the last attention layer, query and key vectors primarily optimize the class token through weighted summation to integrate global information during pretraining—a mechanism specifically designed for the class token but largely irrelevant to the remaining dense tokens. Therefore, it is reasonable to assume that fine-grained local information is preserved in earlier layers but lost in the final layer. To retain such local information within the same feature space, we modify the final layer so that dense tokens bypass the self-attention computation, yielding

(4) $$\begin{array}{cc} x_{\mathrm{dense}} & ={\hat{x}}_{\mathrm{dense}}+\mathrm{MLP}({\hat{x}}_{\mathrm{dense}})\\ \; & =X^{L-1}+X^{L-1}W_{V}^{L}+\mathrm{MLP}(X^{L-1}+X^{L-1}W_{V}^{L}). \end{array}$$

To achieve concept-level image–text alignment, we treat the feature map from the second to last layer of the CLIP image encoder as a collection of *N* image patches, denoted $\left\{e_{\mathrm{patch}}^{1},e_{\mathrm{patch}}^{2},\ldots ,e_{\mathrm{patch}}^{N}\right\}$. For each patch, we compute an initial concept score as

(5) $$s_{i}^{c}=S_{\cos}\left(e_{\mathrm{patch}}^{i},c\right),$$

where *i* indexes the image patch, *c* denotes a textual concept, and *C* is the number of concepts.

**Figure 3:**
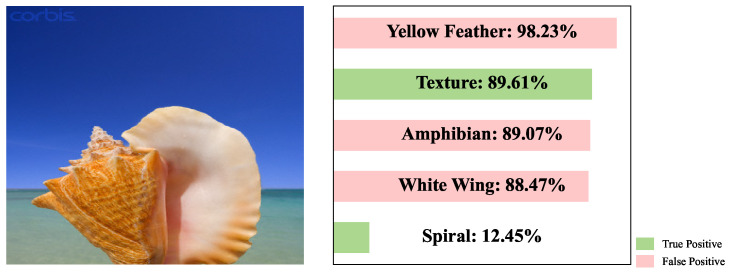
Schematic illustration of initial concept scores.

As shown in Figure 3, the distribution of these initial concept scores still exhibits substantial noise. Notably, concepts clearly absent from the image receive relatively high initial scores. This indicates that although dense tokens enable richer region-level concept representation, they are highly susceptible to noise, often assigning inflated scores to irrelevant concepts and leading to an excessive number of false positives in concept prediction.

Ru et al. [41] observed that the multihead self-attention mechanism in ViT often captures inaccurate feature associations, particularly in deep layers where attention may be diverted by irrelevant regions, leading to low-confidence correlations. This phenomenon helps explain the concept prediction errors that arise when matching image regions to textual concepts using dense tokens. To address this issue, we introduce a training-free concept score refinement pipeline composed of three sequential components: the DMCSR module, the L2GCR module, and the SCCM module. This pipeline mitigates the concept prediction problem, and each component is described in detail below.

#### 2.2.3. Dual Masking Guided Concept Score Refinement Module (DMCSR)

To ensure cross-layer consistency of attention weights, reduce noise interference across layers, and suppress contributions from concept-irrelevant image patches, we propose a dual masking strategy that filters low-confidence information in two steps to refine concept scores.

First, we generate a consistency mask $M_{\mathrm{consis}}$ to retain attention connections with high cross-layer consistency, defined as

(6) $$M_{\mathrm{consis}}(i,j)=1\;\mathrm{if}\;\sum_{l=1}^{L}I(A(i,j,l)>{\bar{A}}_{l})\geq T,$$

where A(i,j,l) denotes the attention weight at position (i,j) in layer *l*, ${\bar{A}}_{l}=\frac{1}{N^{2}}\sum_{i,j}A(i,j,l)$ is the average attention weight in layer *l*, I is the indicator function, and *T* is a hyperparameter. Specifically, for each position (i,j), the mask counts how many layers exhibit an attention weight above the layer-specific mean ${\bar{A}}_{l}$. If this count meets or exceeds the threshold *T*, the position is marked as reliable, i.e., $M_{\mathrm{consis}}(i,j)=1$.

Using this mask, we define the preliminary refined concept consistency score as

(7) $$S_{\mathrm{consis}}(i,c)=\frac{1}{\left|V\right|}\sum_{l\in V}M_{\mathrm{consis}}⊙A_{l}\cdot S_{\mathrm{initial}}(i,c),$$

where V denotes the set of selected layers.

Second, we introduce a concept relevance mask to filter out image patches with weak association to a specific concept. For each concept *c*, we compute its average consistency score across all patches as ${\bar{S}}_{\mathrm{consis}}(c)=\frac{1}{N}\sum_{i=1}^{N}S_{\mathrm{consis}}(i,c)$. If the consistency score of a patch *i* for concept *c* falls below this average, its contribution is discarded. The resulting concept relevance mask is defined as

(8) $$M_{\mathrm{concept}}(i,c)=0\;\mathrm{if}\;S_{\mathrm{consis}}(i,c)<{\bar{S}}_{\mathrm{consis}}(c).$$

By combining the consistency mask and the concept relevance mask, we sequentially select attention connections with strong cross-layer consistency and image regions highly relevant to each concept. This two-stage filtering refines the initial concept scores for each image patch. The output of the DMCSR module, referred to as the refined concept score, can be expressed in terms of the initial concept score as

(9) $$S_{\mathrm{ref}}(i,c)=\frac{1}{\left|V\right|}\sum_{l\in V}M_{\mathrm{consis}}⊙A_{l}\cdot S_{\mathrm{initial}}(i,c)⊙M_{\mathrm{concept}}(i,c).$$

#### 2.2.4. Local-to-Global Concept Reidentification Strategy (L2GCR)

The DMCSR module yields a refined concept score distribution for each image patch. However, these patch-level scores alone cannot reliably reflect the true presence of concepts in the image for two reasons. First, the large number of image patches may lead to spurious high scores for a concept in isolated patches due to random activation fluctuations. Second, many concepts are expressed not only in fine-grained local regions but also through the dominant global structure of the image. To address this, we propose a local-to-global concept reidentification strategy that better captures the genuine activation of each concept, producing a reidentified concept score.

Starting from the refined concept scores $S_{\mathrm{ref}}$, for each concept *c*, we select the top *K* image patches with the highest scores to mitigate the influence of accidental high activations in isolated regions. The local concept score is then computed as the average over these *K* patches:

(10) $$S_{\mathrm{local}}(c)=\frac{1}{K}\sum_{k=1}^{K}\max_{i\in T}S_{\mathrm{ref}}(i,c),$$

where T denotes the set of the top *K* image patches with the highest scores for concept *c*.

To preserve the holistic characteristics, we further compute a global concept score based on $S_{\mathrm{ref}}$. Specifically, for concept *c*, we identify the top *K* highest scoring patches and determine the smallest bounding box that contains all of them. This bounding box defines a concept-specific region, denoted $x_{c}$. In our implementation, we set $K=\sqrt{N}$. Using the original CLIP image encoder and text encoder, denoted $\Phi_{I}$ and $\Phi_{T}$ respectively, we compute the global concept score as the cosine similarity between the cropped region and the textual concept:

(11) $$S_{\mathrm{global}}(c)=S_{\cos}(\Phi_{I}(x_{c}),\Phi_{T}\left(c\right)).$$

To comprehensively capture the importance of concept *c* in the image, we fuse the local and global scores into a reidentified concept score:

(12) $$S_{\mathrm{reid}}(c)=\alpha S_{\mathrm{local}}(c)+\left(1-\alpha \right)S_{\mathrm{global}}(c),$$

where α is a balancing coefficient between the local and global components.

By integrating these two complementary signals, our method explicitly leverages the CLIP image encoder to represent holistic concepts such as shape and color while simultaneously exploiting dense tokens to capture region-level fine-grained concept representations. This joint optimization yields a more accurate and balanced concept score distribution, thereby providing a stronger foundation for the subsequent mapping from concepts to class labels.

#### 2.2.5. Similar Concepts Correction Mechanism (SCCM)

Existing CBMs have been optimized at multiple stages to enhance both classification accuracy and interpretability. Yet, they still face difficulties in discriminating between highly similar concepts that differ only in subtle descriptive attributes, such as “curved beak” versus “long straight beak”. In fine-grained recognition tasks, these nuanced distinctions are often decisive for accurate class separation. Failing to resolve such semantic ambiguities undermines potential improvements in both predictive performance and explanatory clarity. Prior studies have attempted to address classification errors either by directly editing classifier weights [22,24] or by heuristically adjusting concept scores [21,26]. However, these solutions are limited in generalizability, demand case-by-case manual intervention, and rely heavily on expert knowledge for post-hoc corrections.

To address this limitation, we propose an SCCM that integrates Grounding DINO [30] and CLIP to automatically correct mispredictions among similar concepts, thereby enabling more accurate concept activation.

We leverage Grounding DINO’s open vocabulary detection capability and CLIP’s cross-modal similarity computation to recompute scores within groups of similar concepts. The resulting refined scores replace erroneous entries in the reidentified concept scores from the L2GCR module, mitigating CLIP’s weakness in understanding adjective-level distinctions in regional concepts. The detailed procedure is outlined in Algorithm 1 and consists of four main steps.
**Algorithm 1** Similar concepts score recomputation
Given an input image *I*, a concept set *S*, and a temperature coefficient τ, the algorithm outputs the reidentified similarity scores ${\left\{s_{j}\right\}}_{j=1}^{K_{n}}$ for each similar concept group. 1:
**for** each noun n∈N **do** 2:     Construct similar concept group $C_{n}^{s}\leftarrow \left\{\left(a,n\right)\in S\right\}$ 3: **end for** 4: **for** each similar concept group $C_{n}^{s}={\left\{c_{j}\right\}}_{j=1}^{K_{n}}$ **do** 5:     **for** each concept $c_{j}=\left(a_{j},n_{j}\right)\in C_{n}^{s}$ **do** 6:       Generate text prompts $T\leftarrow {\left\{a_{j}n_{j}\right\}}_{j=1}^{K_{n}}$ 7:       Obtain triplets ${\left\{\left(B_{i},c_{i},s_{i}^{c}\right)\right\}}_{i=1}^{N}\leftarrow \mathrm{GroundingDINO}\;\mathrm{Labeler}\left(I,T\right)$ 8:       Apply NMS to remove redundant boxes: ${\left\{\left(B_{i},c_{i},s_{i}^{c}\right)\right\}}_{i=1}^{N_{s}}\leftarrow \mathrm{NMS}\left({\left\{\left(B_{i},c_{i},s_{i}^{c}\right)\right\}}_{i=1}^{N}\right)$ 9:     **end for** 10: **end for** 11: **for** each candidate region $\left(B_{i},c_{i},s_{i}^{c}\right)$ **do** 12:     Crop region corresponding to concept: $R_{i}\leftarrow \mathrm{Crop}\left(I,B_{i}\right)$ 13:     Compute region concept similarity: $s_{i}^{r}\leftarrow \cos\left(E_{\mathrm{img}}\left(R_{i}\right),E_{\mathrm{text}}\left(c_{i}\right)\right)$ 14:     **if** $N_{s}>K_{n}$ **then** 15:       For concepts with multiple regions, compute average similarity
16:       $S^{r}\leftarrow {\left\{s_{i}^{r}\right\}}_{i=1}^{K_{n}}$ 17:       $S^{c}\leftarrow {\left\{s_{i}^{c}\right\}}_{i=1}^{K_{n}}$ 18:     **end if** 19: **end for** 20: **for** each concept group $C_{n}^{s}$ **do** 21:     **for** each concept $c_{j}\in C_{n}^{s}$ **do** 22:       ${\tilde{s}}_{j}\leftarrow \sqrt{s_{j}^{c}\cdot s_{j}^{r}}$ 23:       $s_{j}\leftarrow \frac{\exp({\tilde{s}}_{j}/\tau )}{\sum_{k=1}^{K_{n}}\exp\left({\tilde{s}}_{k}/\tau \right)}$ 24:     **end for** 25: **end for** 26:
**return** ${\left\{s_{j}\right\}}_{j=1}^{K_{n}}$


First, we identify similar concepts within the concept bottleneck. We extract all concepts that follow an adjective–noun format and denote them as S={(a,n)∣a∈A,n∈N}, where A is the set of adjectives and N is the set of nouns. Two concepts $c_{i}=\left(a_{i},n_{i}\right)$ and $c_{j}=\left(a_{j},n_{j}\right)$ are considered similar if they share the same noun ($n_{i}=n_{j}$) but have different adjectives ($a_{i}\neq a_{j}$). For each noun *n*, we define its corresponding similar concept group as $C_{n}^{s}=\left\{\left(a,n\right)\in S\right\}$.

Second, we generate candidate regions for each similar concept group. For a group $C_{n}^{s}={\left\{c_{n_{j}}\right\}}_{j=1}^{K_{n}}$ containing $K_{n}$ concepts, we apply Grounding DINO to perform zero-shot inference on the original image *I*, yielding region proposals for each textual concept. Each proposal includes a pseudo bounding box $B_{i}$, the associated concept $c_{i}$, and a confidence score $s_{i}^{c}$, expressed as

(13) $${\left\{\left(B_{i},c_{i},s_{i}^{c}\right)\right\}}_{i=1}^{N}=\mathrm{GroundingDINO}\;\mathrm{Labeler}\left(I,T\right).$$

As an open vocabulary object detector, Grounding DINO typically produces multiple boxes per concept. We apply Non-Maximum Suppression (NMS) [42] to retain only the top matching boxes for each concept, resulting in a refined set ${\left\{\left(B_{i},c_{i},s_{i}^{c}\right)\right\}}_{i=1}^{N_{s}}$.

Third, we compute region–concept similarity scores. Due to the subword attention effects in Grounding DINO, its confidence scores may overemphasize the noun component and under-represent adjective distinctions. Conversely, CLIP lacks sensitivity to region-level attributes. To combine their strengths, we crop the image *I* into $N_{s}$ regions using the bounding boxes ${\left\{B_{i}\right\}}_{i=1}^{N_{s}}$, where each region corresponds to a concept $c_{i}$ and confidence $s_{i}^{c}$. These cropped regions and their associated textual concepts are fed into CLIP’s image and text encoders, respectively, to compute region–concept cosine similarities. For each concept, if multiple regions are detected, we average their CLIP similarity scores, yielding a refined similarity vector $S^{r}={\left\{s_{i}^{r}\right\}}_{i=1}^{K_{n}}$.

Fourth, we normalize the corrected scores within each similar concept group. Using the Grounding DINO confidence scores from step two, we compute the average confidence per concept to obtain $S^{c}={\left\{s_{i}^{c}\right\}}_{i=1}^{K_{n}}$. We then fuse the two signals by defining the reidentified similarity score as ${\tilde{s}}_{i}=\sqrt{s_{i}^{c}\cdot s_{i}^{r}}$. Recognizing that discriminative attributes should exhibit exclusivity (i.e., one concept should dominate within a similar group), we apply a Softmax function with temperature τ to sharpen the distribution:

(14) $$s_{i}=\frac{e^{{\tilde{s}}_{i}/\tau }}{\sum_{j=1}^{K_{n}}e^{{\tilde{s}}_{j}/\tau }},$$

where τ denotes the temperature coefficient and is set to 0.01, consistent with common practice in CLIP-based similarity scoring, to enhance separation among similar concepts.

After applying this score reallocation to all similar concept groups, we replace the corresponding entries in the reidentified concept scores $S_{\mathrm{reid}}$ with the corrected values, producing the final concept scores $S_{\mathrm{final}}$ for downstream classification.

#### 2.2.6. Interpretable Mapping from Concepts to Classes

The training-free concept score refinement pipeline partially mitigates the limitation of the original CLIP model in assigning reasonable scores to region-level concepts. By optimizing the initial concept score distribution, it better reflects the true presence of concepts in the image: concepts present in the image receive higher scores, absent concepts receive lower scores, and a clear separation emerges between them.

After obtaining the final concept scores $S_{\mathrm{final}}$, a classifier must be learned to map these concepts to class labels for image classification. Conventionally, this mapping is implemented via a fully connected layer $W_{F}\in R^{M\times C}$, where *M* denotes the number of classes and *C* is the number of concepts. However, following the approach of LF-CBM [24], we train a sparser linear layer that relies only on a small subset of key concepts for the final decision, thereby enhancing interpretability.

The optimization objective for the linear classifier is formulated using the cross-entropy loss:

(15) $$\min_{W_{F},b_{F}}\frac{1}{\left|D\right|}\sum_{(x,y)\in D}L_{\mathrm{ce}}(W_{F}f_{c}(x)+b_{F},y),$$

where *D* is the original dataset, $f_{c}\left(x\right)$ denotes the optimized concept scores extracted from image *x*, $b_{F}$ is the bias term, and *y* is the ground-truth class label.

To further enhance interpretability, we introduce two additional regularization terms: a sparsity loss $L_{\mathrm{sp}}$ and an adversarial loss $L_{\mathrm{adv}}$. The sparsity loss [43] is defined as

(16) $$L_{\mathrm{sp}}=\left(1-\beta \right)\frac{1}{2}∥W_{F}{∥}_{F}+\beta {∥W_{F}∥}_{1,1},$$

where ${∥\cdot ∥}_{F}$ denotes the Frobenius norm, ${∥\cdot ∥}_{1,1}$ represents the element-wise matrix norm, and β is set to 0.99 to enforce strong sparsity.

Additionally, we formulate an adversarial loss to penalize the classifier’s reliance on conflicting concepts. Within the selected concept bottleneck, we compute the similarity between all textual concept embeddings and the embeddings of class names. For each class, we identify a fixed number of conflicting concepts and construct a conflict set $C_{\mathrm{conflict}}=\left\{\left(c_{\mathrm{class}},c_{\mathrm{concept}}\right)\right\}$. The ratio of the number of conflicting concept pairs to the total size of the concept bottleneck is denoted by *r*; we set r=0.1, resulting in a conflict set of size r×M×C. The adversarial loss is then expressed as

(17) $$L_{\mathrm{adv}}=\sum_{\left(i,j\right)}\left|W_{F_{i,j}}\right|,\;\left(c_{i},m_{j}\right)\in C_{\mathrm{conflict}},$$

which suppresses the large weights associated with concept–class pairs deemed semantically inconsistent.

The final classifier is optimized by jointly minimizing the cross-entropy, sparsity, and adversarial losses. The complete objective function is given by

(18) $$L=L_{\mathrm{ce}}+\lambda_{\mathrm{sp}}L_{\mathrm{sp}}+\lambda_{\mathrm{adv}}L_{\mathrm{adv}},$$

where $\lambda_{\mathrm{sp}}$ regulates the sparsity of the final linear layer and $\lambda_{\mathrm{adv}}$ modulates the penalty on conflicting concepts.

## 3. Results and Discussion

### 3.1. Experimental Setup

#### 3.1.1. Experimental Data Preparation

We conduct experiments on the six datasets: CIFAR-10, CIFAR-100, CUB-200, Food-101, Flower-102, and ImageNet-100. Furthermore, we include the UC Merced [44] dataset to evaluate the performance of LGA-CBM in the remote sensing domain.The train-test split follows the official partition files provided by each dataset. All input images are preprocessed identically: each image is first resized so that its longer side equals 256 pixels, then randomly cropped to 224×224 pixels, normalized using the ImageNet mean and standard deviation, and augmented with random horizontal flipping. When computing region–concept similarities via Equation (11), the cropped region $x_{c}$ is processed using the same preprocessing pipeline.

#### 3.1.2. Comparison Methods

To validate the effectiveness of our method, we compare against several state-of-the-art CLIP-based CBMs: CLIP zero-shot [27] directly computes the cosine similarity between the image embedding and the text embeddings of class names without any training, assigning the label with the highest similarity score as the final prediction. Linear Probing [27] uses only the CLIP visual encoder to obtain image representations, which are then utilized to train a linear classifier for label prediction. Notably, this method is typically adopted as a baseline in few-shot learning evaluations and is included here specifically for comparison under few-shot experimental settings. PCBM [22] constructs a concept bank using the ConceptNet [45] knowledge graph, treating classes as nodes and incorporating their neighboring nodes as concepts. By expanding multiple hops, it includes more concepts and maps visual embeddings to classes via residual connections. LF-CBM [24] utilizes GPT-3 to generate semantic concepts and employs CLIP as a concept annotator to compute a similarity matrix, thereby supporting flexible integration with arbitrary visual backbone networks. LaBo [25] also leverages GPT-3 to produce candidate concepts but selects a fixed number of concepts per class to form a balanced concept bottleneck. LM4CV [26] adopts a learnable concept search strategy to identify a compact and task-relevant set of concepts from the data, substantially reducing the bottleneck size and yielding more concise and human-understandable explanations. VLG-CBM [46] introduces a vision–language guided framework that leverages Grounding DINO to automatically generate visually grounded concept annotations, enhancing both the faithfulness of concept prediction and the interpretability of model decisions. All compared methods are implemented using the CLIP-ViT-B/32 architecture.

#### 3.1.3. Evaluation Protocol

Classification accuracy serves as the primary metric for model performance. Since LGA-CBM enhances interpretability through a sparse linear layer, we also employ the Number of Effective Concepts (NEC) metric introduced in VLG-CBM to assess interpretability. NEC quantifies how many concepts actively contribute to the final prediction, facilitating comparison of classification accuracy under the constraint of using only a small Number of Effective Concepts.

#### 3.1.4. Implementation Details

All models utilize CLIP-ViT-B/32 as the backbone network. Concept embeddings are computed using the pretrained CLIP weights without fine-tuning. Since none of the compared CBMs have been previously evaluated under the CLIP-ViT-B/32 architecture, we re-implement all methods using the official code available on GitHub and report results on all six datasets. For consistency, concept pools are constructed using the official concepts provided in each original work. For datasets not originally tested, we adhere to the concept generation and filtering procedures described in the respective papers. All algorithms, including the proposed LGA-CBM, are evaluated under identical training settings. Experiments are implemented in PyTorch 1.11.0 and executed on an RTX 4090 Ti GPU under Ubuntu 22.04.

#### 3.1.5. Parameter Settings

Training is performed using the Adam optimizer with an initial learning rate of $1\times 10^{-4}$ and weight decay of $1\times 10^{-5}$. Since pretrained CLIP weights are used, image embeddings are precomputed and cached to accelerate training. In full data settings, the batch size is set to 256. Under few-shot settings, the batch sizes for 1-shot, 2-shot, 4-shot, 8-shot, and 16-shot scenarios are set to 16, 32, 64, 128, and 256, respectively. For CIFAR-10, due to its small number of classes, the corresponding batch sizes are reduced to 2, 4, 8, 16, and 32. The parameter *K* in Equation (10) and the parameter β in Equation (16) are assigned conventional values as specified in their respective equations. For Equation (18), the sparsity control parameter $\lambda_{\mathrm{sp}}$ is optimized using the GLM-SAGA method [47], which generates a regularization path under varying strengths to modulate model performance at a target NEC. The adversarial loss weight $\lambda_{\mathrm{adv}}$, which regulates the classifier’s reliance on conflicting concepts, is fixed at 0.1. Remaining hyperparameters are determined through grid search.

### 3.2. Experimental Results

This section benchmarks the proposed method against state-of-the-art CBMs and includes CLIP zero-shot as a reference baseline. Under few-shot settings, the proposed approach is further compared with the black box linear probe method. All reported metrics are averaged over five independent runs with different random initializations, evaluated at the end of training across all tasks on six datasets. The detailed comparison and analysis are presented as follows.

#### 3.2.1. Classification Accuracy

As shown in Table 1, the proposed method achieves strong performance on image classification across all six datasets, matching or surpassing other state-of-the-art approaches.

In our experiments, LaBo and LM4CV demonstrate comparable performance but exhibit limitations on fine-grained datasets. This stems primarily from the lack of concept length control during generation, which introduces substantial noise into the concept bottleneck. Although their bottleneck size approaches the visual embedding dimension, enabling decent accuracy on coarse datasets, the accumulated noise severely degrades feature quality for fine-grained classification. In contrast, LF-CBM and VLG-CBM employ more refined concept selection and train sparse linear classifiers that achieve high accuracy using only a small number of concepts. The proposed LGA-CBM further improves concept prediction by fusing local and global concept representations, providing superior features for sparse classifier training. Consequently, LGA-CBM achieves the highest classification accuracy on five of the six datasets, CIFAR-10, CIFAR-100, CUB-200, Flower-102, and ImageNet-100, outperforming the second-best method by 0.99%, 2.42%, 2.27%, 0.85%, and 0.33%, respectively. On Food-101, it attains the second-best accuracy.

#### 3.2.2. Few-Shot Performance

**Figure 4:**
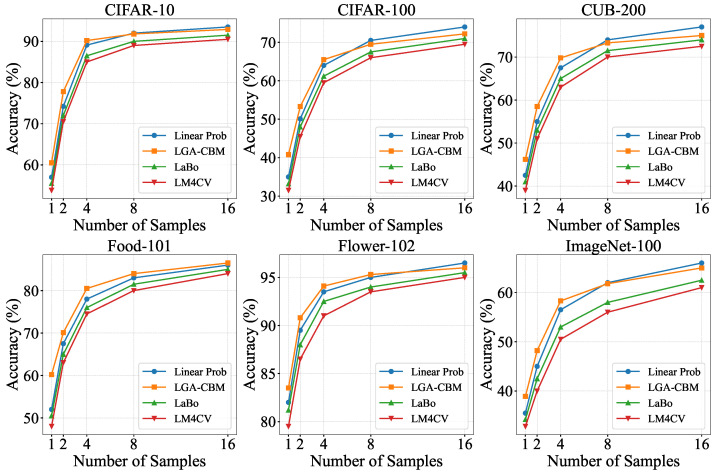
Classification accuracy comparison under few-shot settings.

We further evaluate LGA-CBM under varying few-shot settings (1-shot to 16-shot). As illustrated in Figure 4, comprehensive assessments are conducted across all six datasets.

The results indicate that LGA-CBM consistently outperforms both LaBo and LM4CV across all few-shot scenarios and often surpasses the standard linear probe. Notably, under 1-shot, 2-shot, and 4-shot settings, LGA-CBM improves average accuracy by 3% to 7% across the six datasets, demonstrating its effectiveness in data-scarce regimes. This robust few-shot performance stems from the capacity of LGA-CBM to produce consistent responses to key concepts through local-to-global perception. Black-box few-shot methods suffer from overfitting due to limited samples and pixel-level noise, leading to poor generalization. In contrast, LGA-CBM leverages the pretrained image–text encoders of CLIP to ground textual concepts in image regions, enabling the learning of an interpretable classifier even with few examples.

#### 3.2.3. Few-Shot Performance on Remote Sensing Dataset

As shown in Table 2, we evaluate LGA-CBM on the UC Merced dataset under few-shot settings to verify its effectiveness in remote sensing applications. The results demonstrate that LGA-CBM consistently achieves superior or competitive performance across all shot configurations, validating the transferability of our training-free refinement pipeline to domain-shifted scenarios.

Specifically, LGA-CBM obtains the best accuracy in 1-shot to 8-shot settings, outperforming the second-best method by significant margins. This improvement stems from the DMCSR module suppressing background noise and the L2GCR strategy harmonizing local and global cues. Notably, the performance gap is most pronounced in the 1-shot setting. In contrast, as shots increase to 16, the Linear Probe baseline slightly surpasses LGA-CBM. This trend aligns with expectation since supervised parameter optimization on visual features can effectively adapt to the target domain distribution when sufficient labeled samples are available. Nevertheless, LGA-CBM maintains advantage over other interpretable CBMs. The absolute accuracy levels are moderately lower than natural image benchmarks such as CIFAR-10 and Flower-102, which reasonably reflects the domain gap between CLIP pretraining data which are primarily natural photographs and the overhead perspective of remote sensing imagery. Despite this gap, the LGA-CBM consistent improvement margins over the baselines underscore that our local–global awareness design is fundamentally domain-agnostic. The attention-guided masking and region–text alignment operations function on architectural properties of ViT rather than dataset-specific statistics.

In summary, the UC Merced experiments substantiate that LGA-CBM effectively mitigates CLIP region-level perception limitations, enabling reliable and interpretable classification even under significant domain shift. These findings demonstrate that LGA-CBM is well-suited for sensor-driven applications where localized visual evidence and transparent reasoning are paramount.

#### 3.2.4. Classification Accuracy Under Varying Numbers of Effective Concepts

We further compare methods under different NECs. Since LF-CBM, VLG-CBM, and LGA-CBM use sparse linear layers to map concepts to classes, their sparsity is controlled by adjusting $\lambda_{\mathrm{sp}}$ in Equation (18). For each target NEC, we select the weight matrix closest to the desired sparsity and prune weights with the smallest absolute values to enforce exact NEC alignment. For LM4CV, the NEC is controlled by selecting fewer concepts during training. As a baseline, we also construct a random concept bottleneck of size 512 and apply the same pruning procedure.

**Figure 5:**
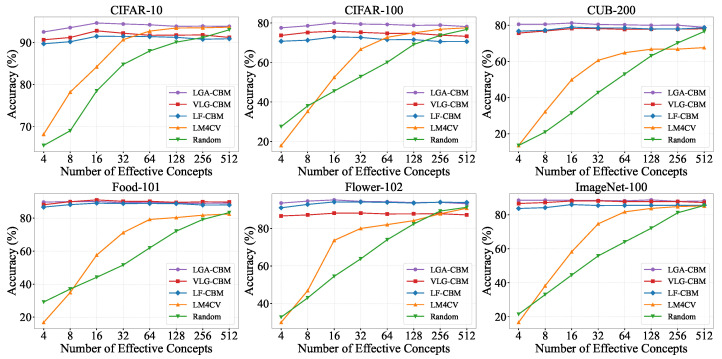
Classification accuracy comparison under different NECs.

As shown in Figure 5, even randomly constructed concept bottlenecks achieve near-optimal accuracy when NEC equals 512, confirming the theoretical finding in VLG-CBM [46]. However, as NEC decreases, the accuracy of the random and LM4CV methods drops sharply due to aggressive pruning. In contrast, the three sparse training methods degrade more gracefully, indicating that their weight matrices effectively associate discriminative concepts with correct classes, thereby rendering them resilient to pruning. Notably, LGA-CBM exhibits a clear advantage at very low NEC values. This advantage arises because its optimized concept scores provide cleaner, more accurate concept activations, allowing the sparse classifier to focus on truly relevant concepts without being misled by the inherent limitations of CLIP in region-level concept scoring.

#### 3.2.5. Computational Efficiency Analysis

Although LGA-CBM functions as a training-free framework, incorporating the SCCM module necessitates calling Grounding DINO during inference, inevitably incurring additional computational overhead. To assess this trade-off, we performed a comprehensive performance evaluation in terms of FLOPs, memory consumption, and inference latency, with the results summarized in Table 3.

As shown in the table, LGA-CBM incurs higher computational costs than other methods. Specifically, the inference latency reaches 48.75 ms per image, whereas other methods fall within the 10.42–21.98 ms range. Nevertheless, LGA-CBM sustains an inference speed of approximately 20 FPS, which is adequate for many real-world sensor-driven applications, such as environmental monitoring and medical image analysis. While integrating Grounding DINO adds computational overhead to LGA-CBM, the resulting inference efficiency remains within an acceptable range. More importantly, the additional cost yields substantially enhanced interpretability and classification accuracy, which are essential for explainable vision–language classification systems. Therefore, the trade-off between computational cost and interpretability is both reasonable and well justified in many real-world applications.

### 3.3. Ablation Study

This subsection investigates the impact of key components and parameters in concept selection and decision making. All performance metrics are averaged over the six datasets. The results and analysis are as follows.

#### 3.3.1. Impact of Backbone Variations

To evaluate the influence of backbone on the proposed framework, we replace CLIP with its distilled variant TinyCLIP [48] and report the corresponding classification results in Table 4.

The results demonstrate that LGA-CBM consistently surpasses the zero-shot baselines across both backbone architectures. Notably, LGA-CBM effectively mitigates the performance degradation typically associated with model distillation. While the zero-shot performance disparity between CLIP and TinyCLIP exhibits substantial variation across datasets, this gap is markedly attenuated within the LGA-CBM framework. This suggests that our method compensates for the feature diversity loss inherent in distillation by reinforcing cross-layer attention consistency, harmonizing local–global representations, and rectifying mispredictions among semantically similar concepts. Overall, these findings confirm that LGA-CBM reduces dependency on backbone capacity, enabling competitive performance even with lightweight models.

#### 3.3.2. DMCSR Module and L2GCR Strategy

DMCSR introduces the consistency mask and concept relevance mask to refine initial concept scores, followed by the L2GCR strategy. To validate their contributions, we conduct ablation studies summarized in Table 5, where metrics are averaged across all six datasets.

When only global concept scores are utilized, the method reverts to the original CLIP scoring approach and yields the lowest accuracy across all settings. Incorporating the consistency mask to select cross-layer consistent attention connections and fusing the resulting scores with global scores improves accuracy by more than 1%. Further applying the concept relevance mask enables the model to identify regions most relevant to each concept, thereby extracting more discriminative local features and achieving the highest accuracy. Conversely, removing the global component and relying solely on mask-refined local scores leads to a performance drop, indicating that despite CLIP’s insensitivity to concept-level alignment, the initial global scores still capture useful holistic information, particularly for attributes like color and shape that span the entire image. This confirms the effectiveness of the proposed L2GCR Strategy.

#### 3.3.3. Attention Layer Set V

**Figure 6:**
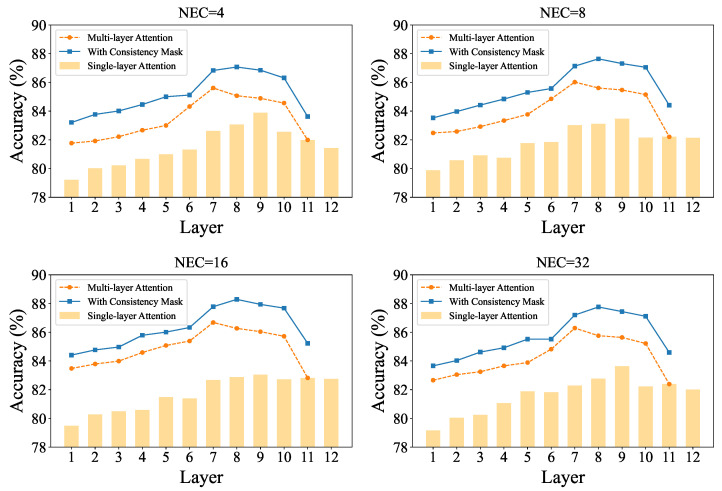
Classification accuracy comparison under different settings of the attention layer set V.

The previous experiment confirmed the effectiveness of the proposed concept score refinement pipeline, particularly the step employing the consistency mask $M_{\mathrm{consis}}$ to compute preliminary concept consistency scores. To evaluate the impact of the attention layer set V in Equation (7), we conduct experiments with |V|=1 and |V|>1. The results are shown in Figure 6. The yellow bars represent using a single-layer’s attention weights to compute the average, the orange curve corresponds to averaging attention weights from layer *i* to layer 11, and the blue curve further applies the consistency mask $M_{\mathrm{consis}}$ derived from Equation (6) to refine the initial concept scores.

Experiments under varying NECs demonstrate that the method incorporating the consistency mask consistently outperforms both the multi-layer average without masking and the single-layer approach. By adjusting the starting layer, we observe that utilizing layers 8 to 11 yields the highest classification accuracy. This suggests that layers 8 to 11 in CLIP-ViT encode highly semantic concept representations. When the starting layer is lowered below layer 7, performance declines due to interference from low-level visual features that degrade mask quality. Conversely, when the starting layer exceeds layer 9, the reduced number of layers in V leads to higher variance in the averaged attention weights, thereby impairing mask reliability. Therefore, in practice, we set V={8,9,10,11} and fix the threshold T=3 in Equation (6) to suppress noise from individual attention weights.

Further analysis of the no-mask setting reveals that among single-layer choices, layer 9 yields the best performance, indicating that its visual features exhibit strong concept representativeness. We hypothesize that prior to layer 9, high-level semantic concepts are not fully formed, while beyond layer 9, CLIP begins extracting increasingly abstract representations where regional details tend to become blurred. By the final layer, the class token dominates the embedding, shifting focus toward global category cues. When averaging multiple layers without masking, the best performance is achieved using layers 7 to 11, suggesting that incorporating more layers helps mitigate layer-specific noise in the absence of consistency guidance. However, even this multi-layer average cannot match the performance of the consistency-masked approach, underscoring the superiority of the proposed mask.

#### 3.3.4. Components in SCCM

**Figure 7:**
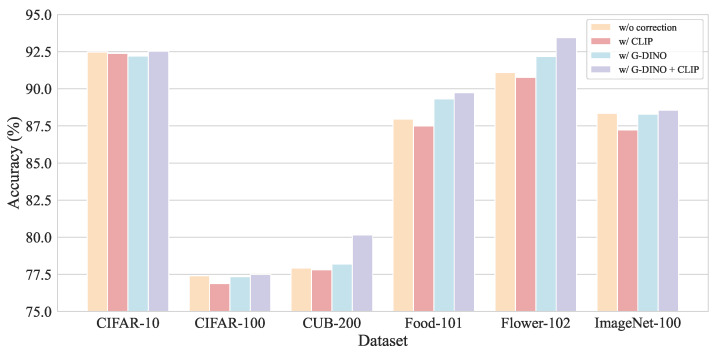
Classification accuracy comparison under different concept correction methods.

To validate the contribution of SCCM, we evaluate three variants under an effective concept count of 4: using CLIP alone, Grounding DINO alone, and the combined CLIP and Grounding DINO approach. All variants employ the normalization in Equation (14). Results are shown in Figure 7.

On CIFAR-10, CIFAR-100, and ImageNet-100, the correction mechanism provides minimal improvement because these coarse datasets contain few similar concept pairs. In contrast, on the fine-grained datasets CUB-200, Food-101, and Flower-102, where the concept bottleneck includes many adjective–noun concept pairs and CLIP struggles with region-level distinctions, the automated correction significantly boosts accuracy.

Comparing the two ablated versions, the full SCCM outperforms both. Using CLIP alone for correction actually degrades performance relative to the base refinement module, as CLIP lacks fine-grained discriminative power. Grounding DINO alone exhibits a bias toward nouns, often overlooking adjective-modified distinctions. The proposed combination addresses these issues: Grounding DINO localizes relevant regions, and CLIP computes fine-grained similarities within those crops. Softmax normalization then amplifies subtle differences between similar concepts, enabling more accurate score reallocation.

#### 3.3.5. Weight Coefficient α

**Figure 8:**
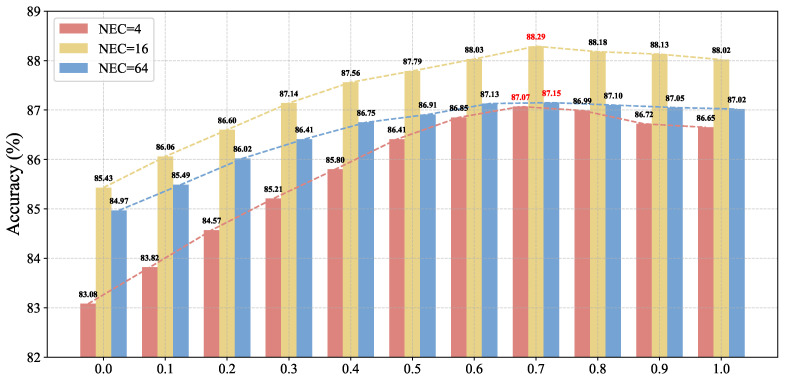
Classification accuracy comparison under different values of the weight coefficient α. The red values indicate the peak performance for each setting.

As shown in Figure 8, We evaluate the influence of the fusion weight α under effective concept counts of 4, 16, and 64, using average accuracy across six datasets as the metric.

The results reveal that peak performance occurs at α=0.7 in all settings, indicating optimal balance between local and global cues. When α<0.7, increased reliance on global scores diminishes the contribution of local features, degrading performance on fine-grained datasets and lowering overall accuracy. At α=0, the model depends entirely on CLIP’s global scores, losing region awareness and resulting in significant degradation in concept prediction. Conversely, when α>0.7, excessive emphasis on local patches overlooks globally salient concepts such as red or circular, which CLIP captures effectively. This imbalance also harms performance.

The sensitivity to α varies with concept count. With fewer concepts (e.g., 4 or 16), each concept exerts stronger influence on the final logits, making accuracy highly sensitive to α. With more concepts (e.g., 64), the decision is distributed across many concepts, dampening the impact of any single concept’s score and reducing sensitivity to α.

#### 3.3.6. Loss Terms

To validate the effectiveness of the interpretable mapping objective, we evaluate the contribution of each loss component under varying effective concept counts. Results are summarized in Table 6.

Employing solely cross-entropy loss $L_{\mathrm{ce}}$ yields the poorest performance, particularly with limited concepts (e.g., 4 or 8), as the classifier lacks structural priors to identify discriminative concepts and tends to overfit or rely on redundant features. Incorporating adversarial loss $L_{\mathrm{adv}}$ enhances results, confirming that penalizing semantically conflicting concept–class associations enhances robustness and discriminability. Integrating sparsity loss $L_{\mathrm{sp}}$ without $L_{\mathrm{adv}}$ produces substantial gains, attaining second-best performance across all settings. This indicates that $L_{\mathrm{sp}}$ effectively directs the model to prioritize a few key concepts, mitigating redundancy while enhancing interpretability. The full loss combination achieves optimal performance universally, affirming the complementarity of sparsity and adversarial regularization: the former fosters interpretability by enforcing concept economy, while the latter mitigates semantic interference. Notably, $L_{\mathrm{adv}}$ offers greater gains when the concept count is large. With few concepts, the model naturally concentrates on key features even without explicit conflict penalties. With many concepts, however, redundant or conflicting concepts are more prone to misuse, and $L_{\mathrm{adv}}$ plays a critical role in filtering them out, thereby improving both accuracy and explanation quality.

### 3.4. Interpretability

This subsection presents interpretable outputs on test examples to visually illustrate the interpretability of LGA-CBM. The analysis examines two aspects as follows.

#### 3.4.1. Concept Contributions

Visualizing the contribution of different textual concepts to classification decisions provides intuitive insight into the reasoning process of CBMs. Following the definition in LF-CBM, the contribution of the *i*th concept to the *j*th class is given by $\mathrm{Contribution}(i,j)=s_{i}\cdot W_{F}(j,i)$, where $s_{i}$ is the concept score and $W_{F}(j,i)$ is the corresponding classifier weight. Since the linear layer is sparse, most concept contributions are zero, and each class is determined by only a few key concepts. The magnitude of these non-zero contributions can be effectively visualized using bar plots.

**Figure 9:**
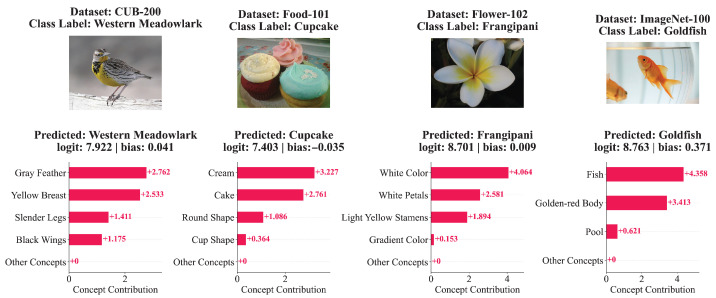
Visualization of concept contributions for random samples across datasets.

Due to the low resolution of images in CIFAR-10 and CIFAR-100, Figure 9 presents concept contribution visualizations only for the remaining four datasets. Setting NEC to 4, many classes are decided by exactly four concepts. For instance, the class Western Meadowlark is determined by the concepts gray feathers, yellow breast, slender legs, and black wings, accurately reflecting distinct visual attributes across different image regions. Moreover, the proposed method captures not only local concepts but also global ones. Examples include circular and cup-shaped for cupcake, white and gradient color for frangipani, and fish-like for goldfish. When using the sparse linear layer for classification, each class relies on only a few concepts, with contributions from all other concepts being exactly zero.

#### 3.4.2. Concept Activation Aligned with Images

**Figure 10:**
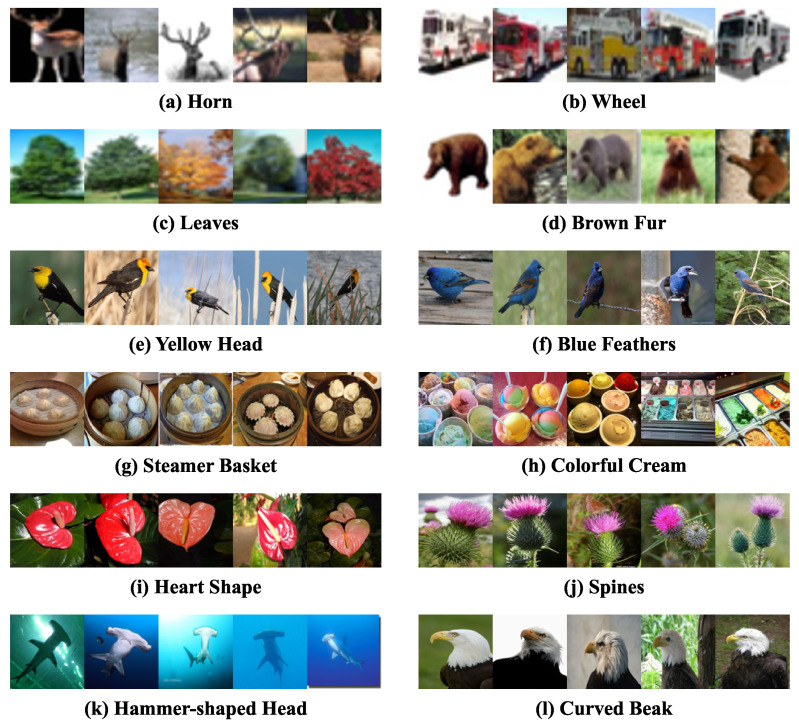
Top five activated images corresponding to each example concept.

To validate whether the learned concepts align with human cognition, we select two textual concepts from the concept bottleneck of each of the six datasets and retrieve the top five images with the highest activation scores for each concept. The results are presented in Figure 10.

The visualization confirms that the proposed method accurately associates image content with human-understandable concepts. First, in local concept recognition, fine-grained structural features such as ‘horn’ and ‘wheel’ are effectively captured. The retrieved high-activation samples clearly exhibit the corresponding local attributes: images under the ‘horn’ concept prominently display animal horns in salient positions, while those under the ‘wheel’ concept show clear wheels of vehicles or machinery occupying significant image regions. This demonstrates the model’s strong capability in perceiving local features and extracting region-specific information even in complex backgrounds.

Second, for global concepts describing overall shape or texture, such as ‘heart-shaped’ and ‘spiky’, the model also achieves accurate modeling. High-activation images consistently exhibit global characteristics matching the concept descriptions: objects under ‘heart-shaped’ display an overall contour resembling a heart, and those under ‘spiky’ feature pronounced spike-like surface structures. This further validates the effectiveness of the proposed method in capturing macro-level visual semantics, demonstrating the model’s ability to balance attention between local details and global morphology.

In summary, the concept activation visualization provides direct evidence that the proposed method not only improves image classification performance but also achieves better alignment with human cognitive patterns at the concept-level, offering strong support for model interpretability.

#### 3.4.3. Visualization of Region-Level Concept Sensitivity

**Figure 11:**
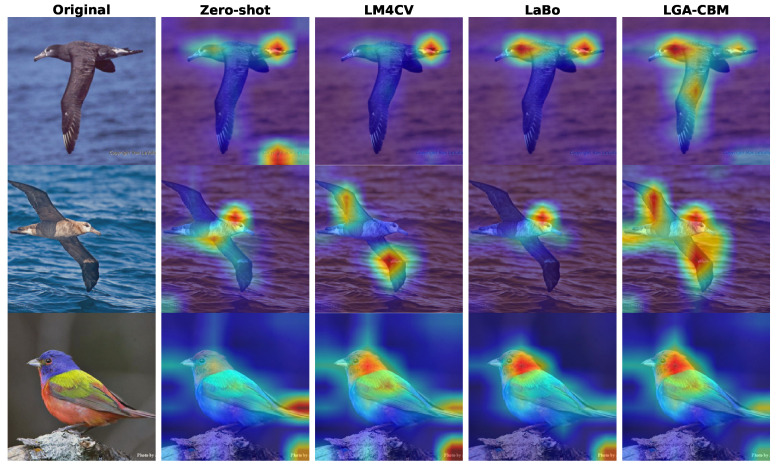
Visualization of attention maps for different methods.

To directly validate the model’s sensitivity to multiple local concepts, we visualize and compare the attention maps of LGA-CBM against Zero-shot CLIP, LM4CV, and LaBo on representative samples from the CUB-200 dataset, as shown in Figure 11.

The visualization reveals distinct differences in region-level perception. Zero-shot CLIP exhibits global representation bias, producing either overly concentrated activations on single regions or diffuse responses that include background noise. LM4CV and LaBo show marginal improvements but still tend to highlight only one dominant region. For instance, in the third row (Painted Bunting), Zero-shot CLIP incorrectly focuses on the background, while LM4CV and LaBo primarily activate the head region, neglecting other discriminative body parts. In contrast, LGA-CBM successfully activates multiple spatially distinct regions corresponding to different semantic concepts. As observed in the first and second rows, our method simultaneously highlights both the head and wings regions, demonstrating enhanced sensitivity to co-occurring local evidence. The attention maps are more compact and semantically aligned with actual object parts, confirming that the proposed DMCSR and L2GCR modules effectively mitigate CLIP’s global bias and improve multi-concept perception capability.

These qualitative results provide direct evidence that LGA-CBM enhances region-level concept sensitivity, complementing the quantitative improvements observed in classification accuracy.

### 3.5. Further Discussion

We propose a Dual Masking Guided Concept Score Refinement method based on the attention weights of CLIP-ViT, partially addressing CLIP’s limited capability in recognizing region-level concepts. Indeed, recent studies [49] have shown that CLIP suffers from additional limitations beyond poor regional feature extraction. Specifically, it struggles to accurately interpret spatial relationships, adjectives, and negation words. These shortcomings stem from CLIP’s pretraining paradigm, which does not enforce fine-grained alignment between image regions and textual elements at these levels of linguistic structure. Therefore, if CLIP continues to serve as the backbone network for CBMs, targeted improvements must be developed to refine concept score distributions and compensate for these inherent weaknesses.

In addition, the performance of the SCCM module is closely tied to the quality of the external open-vocabulary detector, Grounding DINO. In highly specialized domains, pretrained general-purpose detectors may lack domain-specific conceptual knowledge. In such cases, inaccurate bounding boxes could theoretically lead to error accumulation. To enhance robustness against such failures, SCCM adopts a confidence-weighted fusion strategy (Algorithm 1, Line 22), ensuring that low-confidence detections from Grounding DINO exert minimal influence on the final concept scores. Moreover, since SCCM operates as a refinement layer built upon the robust reidentified concept scores, the framework retains a reliable fallback mechanism even when external detections are unreliable. For domain-specific deployments, Grounding DINO can be fine-tuned on relevant domain datasets or replaced with specialized detectors, leveraging the modular design of LGA-CBM.

At the same time, vision–language models with stronger fine-grained understanding capabilities are rapidly advancing. Models such as Semantic SAM [50], Grounded SAM [51], and FineCLIP [52] have already demonstrated promising performance in extracting fine-grained conceptual information. Future CBMs are likely to harness these emerging multimodal foundation models to further alleviate inaccuracies in concept prediction and strengthen alignment between visual content and human-interpretable semantics.

## 4. Conclusions

We introduce LGA-CBM to enhance the reliability of concept scores in CLIP-based interpretable classification. First, the initial concept scores are computed by leveraging image patch embeddings and a set of candidate textual concepts. Subsequently, we propose a training-free concept score refinement pipeline comprising three components: the DMCSR module, the L2GCR strategy, and the SCCM. This pipeline jointly optimizes the activation strength of textual concepts from both local and global perspectives, yielding more faithful and discriminative concept scores. Finally, a sparse linear layer maps the refined concept scores to class labels, enabling highly interpretable image classification with only a few active concepts. LGA-CBM achieves state-of-the-art classification accuracy across all six benchmark datasets while generating concept-based explanations that align closely with human cognition.

## Figures and Tables

**Table 1:** Classification accuracy (%) of different CBMs.

Method	CIFAR-10	CIFAR-100	CUB-200	Food-101	Flower-102	ImageNet-100
Zero-shot [27]	91.30 *	65.10 *	62.54	84.40 *	66.70 *	74.65
PCBM [22]	81.87	59.61	61.82	N/A	N/A	N/A
LF-CBM [24]	91.49	72.88	78.96	89.10	94.14	86.02
LaBo [25]	93.04	76.70	66.55	83.39	91.27	85.50
LM4CV [26]	93.67 *	77.55 *	67.64 *	82.50 *	90.78 *	85.27
VLG-CBM [46]	92.80	75.82	78.14 *	91.07 *	94.36 *	88.23
**LGA-CBM (Ours)**	**94.66**	**79.97**	**81.23**	90.11	**95.21**	**88.56**

The best results are highlighted in bold, the second-best are underlined, results reproduced from original papers are marked with *, and inapplicable methods are denoted as N/A.

**Table 2:** Classification accuracy (%) on the remote sensing dataset UC Merced.

Method	1-Shot	2-Shot	4-Shot	8-Shot	16-Shot
Linear Prob	58.88	71.05	83.49	87.65	**92.00**
LaBo	57.58	69.49	82.16	86.21	90.17
LM4CV	55.51	67.46	80.29	85.16	89.57
LGA-CBM (Ours)	**62.51**	**74.29**	**84.38**	**88.63**	91.80

The best results are highlighted in bold, and the second-best are underlined.

**Table 3:** Comparison of computational cost.

Method	FLOPs (G)↓	GPU (GB)↓	Latency (ms)↓	CIFAR-100 (%)↑	CUB-200 (%)↑
Zero-shot	**5.91**	**3.43**	**10.42**	65.10	62.54
LM4CV	6.03	4.91	20.45	77.55	67.64
VLG-CBM	6.22	5.37	21.98	75.82	78.14
LGA-CBM	23.59	11.81	48.75	**79.97**	**81.23**

↓ indicates that lower values are better; ↑ indicates that higher values are better. The best results are highlighted in bold, and the second-best are underlined.

**Table 4:** Impact of different backbones on classification accuracy (%).

Method	CIFAR-10	CIFAR-100	Food-101	Flower-102
Zero-shot (CLIP)	91.3 *	65.1 *	84.1 *	66.7 *
Zero-shot (TinyCLIP)	92.2 *	71.8 *	78.3 *	60.7 *
LGA-CBM (CLIP)	**94.7**	80.0	**90.1**	**95.2**
LGA-CBM (TinyCLIP)	94.6	**82.1**	88.2	91.6

The best results are highlighted in bold, the second-best are underlined, results reproduced from original papers are marked with *.

**Table 5:** Impact of different concept score combinations on classification accuracy (%).

$S_{\mathrm{global}}$	$M_{\mathrm{consis}}$	$M_{\mathrm{concept}}$	NEC					
			4	8	16	32	64	128
✓			83.08	83.97	85.43	85.18	84.97	84.78
✓	✓		84.91	85.08	86.33	85.92	85.65	85.53
✓		✓	83.62	84.38	85.71	85.44	84.99	85.10
	✓	✓	86.55	87.24	87.65	87.39	86.81	86.84
✓	✓	✓	**87.07**	**87.64**	**88.29**	**87.76**	**87.15**	**87.35**

The best results are highlighted in bold, and the second-best are underlined.

**Table 6:** Impact of different loss combinations on classification accuracy (%).

$L_{\mathrm{ce}}$	$L_{\mathrm{sp}}$	$L_{\mathrm{adv}}$	NEC					
			4	8	16	32	64	128
✓			36.82	43.17	52.77	60.63	69.51	74.70
✓		✓	37.26	43.55	53.81	62.95	73.96	78.99
✓	✓		86.91	87.11	87.14	85.22	84.08	83.81
✓	✓	✓	**87.07**	**87.64**	**88.29**	**87.76**	**87.15**	**87.35**

The best results are highlighted in bold, and the second-best are underlined.

## Data Availability

The datasets used in this study are publicly available from the following sources: CIFAR-10 and CIFAR-100 [35] at http://www.cs.toronto.edu/~kriz/cifar.html (accessed on 15 March 2024); CUB-200 [28] at https://www.vision.caltech.edu/datasets/cub_200_2011/ (accessed on 15 March 2024); Food-101 [36] at https://data.vision.ee.ethz.ch/cvl/datasets_extra/food-101/ (accessed on 15 March 2024); Flowers-102 [37] at https://www.robots.ox.ac.uk/~vgg/data/flowers/102/ (accessed on 15 March 2024); ImageNet [38] (from which ImageNet-100 is sampled) at https://image-net.org (accessed on 15 March 2024). No new data were created or modified in this work.

## Abbreviations

The following abbreviations are used in this manuscript:

LGA-CBM	Local–Global Aware Concept Bottleneck Model
DMCSR	Dual Masking Guided Concept Score Refinement
L2GCR	Local-to-Global Concept Reidentification
SCCM	Similar Concepts Correction Mechanism
CBMs	Concept Bottleneck Models
NEC	Number of Effective Concepts
